# Determinants of patient-reported outcome trajectories and symptomatic recovery in Improving Access to Psychological Therapies (IAPT) services

**DOI:** 10.1017/S0033291720005395

**Published:** 2022-10

**Authors:** Jan Stochl, Emma Soneson, Freya Stuart, Jessica Fritz, Annabel E. L. Walsh, Tim Croudace, Joanne Hodgekins, Ushma Patel, Debra A. Russo, Clare Knight, Peter B. Jones, Jesus Perez

**Affiliations:** 1 Department of Psychiatry, University of Cambridge, Cambridge, UK; 2 National Institute for Health Research Applied Research Collaboration (ARC) East of England (EoE), Cambridge, UK; 3 Department of Kinanthropology, Charles University, Prague, Czechia; 4 Institution of Psychiatry, Psychology and Neuroscience, King's College London, London, UK; 5 School of Health Sciences, University of Dundee, Dundee, UK; 6 Norwich Medical School, University of East Anglia, Norwich, UK; 7 Cambridgeshire & Peterborough NHS Foundation Trust, Cambridge, UK

**Keywords:** patient-reported outcomes, predictors, symptomatic recovery, psychotherapy, Improving Access to Psychological Therapies

## Abstract

**Background:**

Despite evidence for the general effectiveness of psychological therapies, there exists substantial heterogeneity in patient outcomes. We aimed to identify factors associated with baseline severity of depression and anxiety symptoms, rate of symptomatic change over the course of therapy, and symptomatic recovery in a primary mental health care setting.

**Methods:**

Using data from a service evaluation involving 35 527 patients in England's psychological and wellbeing [Improving Access to Psychological Therapies (IAPT)] services, we applied latent growth models to explore which routinely-collected sociodemographic, clinical, and therapeutic variables were associated with baseline symptom severity and rate of symptomatic change. We used a multilevel logit model to determine variables associated with symptomatic recovery.

**Results:**

Being female, younger, more functionally impaired, and more socioeconomically disadvantaged was associated with higher baseline severity of both depression and anxiety symptoms. Being older, less functionally impaired, and having more severe baseline symptomatology was associated with more rapid improvement of both depression and anxiety symptoms (male gender and greater socioeconomic disadvantage were further associated with rate of change for depression only). Therapy intensity and appointment frequency seemed to have no correlation with rate of symptomatic improvement. Patients with lower baseline symptom severity, less functional impairment, and older age had a greater likelihood of achieving symptomatic recovery (as defined by IAPT criteria).

**Conclusions:**

We must continue to investigate how best to tailor psychotherapeutic interventions to fit patients’ needs. Patients who begin therapy with more severe depression and/or anxiety symptoms and poorer functioning merit special attention, as these characteristics may negatively impact recovery.

## Introduction

Evidence abounds for the effectiveness of psychological therapies in treating a wide range of mental health problems (Barth et al., 2013; Cristea et al., 2017; Cuijpers, Cristea, Karyotaki, Reijnders, & Huibers, 2016; Cuijpers et al., 2014a; 2014b; Lambert, 2013). However, it is widely acknowledged that treatment outcomes vary greatly between individuals with a significant proportion not responding at all (Van, Dekker, Peen, Van Aalst, & Schoevers, 2008a; Van et al., 2008b). The ability to explain why individuals respond differently to therapy provides important supplementary information to advance our understanding of ‘what works for whom’ (Green et al., 2015; Van et al., 2008a). Characterising the basis of heterogeneity in baseline symptom severity, rates of symptomatic change during therapy, and treatment outcomes enables us to identify variables related to treatment success and thus may constitute a step towards more personalised care. Furthermore, provision of appropriate, tailored treatment enables efficient allocation of limited mental health resources.

Much effort has gone into understanding the reasons for variation in therapy response. At the patient level alone, more than 200 factors have been proposed to potentially influence therapy outcomes (Norcross & Wampold, 2011). These variables include sociodemographic characteristics [e.g. age (Amati, Banks, Greenfield, & Green, 2018; Marttunen, Välikoski, Lindfors, Laaksonen, & Knekt, 2008; Robinson, Kellett, & Delgadillo, 2020; Wolitzky-Taylor, Arch, Rosenfield, & Craske, 2012), gender (Amati et al., 2018; Cuijpers et al., 2014a, 2014b; Wolitzky-Taylor et al., 2012), socioeconomic status (Amati et al., 2018; Green et al., 2015; Marttunen et al., 2008), and ethnicity (Amati et al., 2018; Green et al., 2015; Robinson et al., 2020; Saxon, Firth, & Barkham, 2017; Wolitzky-Taylor et al., 2012)], mental health-related clinical variables [e.g. pre-treatment disorder severity (Amati et al., 2018; Green et al., 2015; Gyani, Shafran, Layard, & Clark, 2013a; 2013b; Marttunen et al., 2008; Robinson et al., 2020; Saxon et al., 2017; Van et al., 2008a; Wolitzky-Taylor et al., 2012), comorbidities (Amati et al., 2018; Goddard, Wingrove, & Moran, 2015; Marttunen et al., 2008; Wolitzky-Taylor et al., 2012), and psychiatric history (Marttunen et al., 2008)], social functioning and support (Amati et al., 2018; Lindfors, Ojanen, Jääskeläinen, & Knekt, 2014; Wang, Mann, Lloyd-Evans, Ma, & Johnson, 2018), and personality traits (Bucher, Suzuki, & Samuel, 2019; Laaksonen, Knekt, & Lindfors, 2013a; Laaksonen, Knekt, Sares-Jäske, & Lindfors, 2013b). Outcomes further vary by treatment variables [e.g. therapy modality (Amati et al., 2018; Gyani et al., 2013a, 2013b; Marttunen et al., 2008), number of sessions attended (Amati et al., 2018; Gyani et al., 2013a, 2013b; Norcross & Wampold, 2011) and missed (Amati et al., 2018; Green et al., 2015; Van et al., 2008a), time waited to start treatment (Clark et al., 2018), therapy setting (Amati et al., 2018), frequency of sessions (Tiemens et al., 2019), therapeutic alliance (Del Re, Flückiger, Horvath, Symonds, & Wampold, 2012; Horvath, Del Re, Flückiger, & Symonds, 2011), treatment engagement (Dixon, Holoshitz, & Nossel, 2016), and patient expectations of therapy outcome (Porter & Chambless, 2015)] as well as on therapist characteristics and experience (Amati et al., 2018; Gyani et al., 2013a, 2013b; Nissen-Lie, Monsen, Ulleberg, & Rønnestad, 2013). Although some of these factors influence treatment outcome in a consistent direction [good therapeutic alliance, for example, consistently leads to more positive outcomes (Del Re et al., 2012; Horvath et al., 2011)], several show inconclusive evidence regarding direction of effect (Amati et al., 2018).

Individuals may further vary in their progression through therapy, leading to heterogeneity in treatment response trajectories (Green et al., 2015). These trajectories are informative for understanding progress as well as predicting outcomes (Comninos & Grenyer, 2007; Lutz, Stulz, & Kock, 2009; Lutz et al., 2014; Stulz, Lutz, Leach, Lucock, & Barkham, 2007). Research has aimed to characterise differences in treatment response trajectories, largely through class-based approaches that classify sub-groups of patients with homogeneous treatment response trajectories and determine predictors of group membership. These studies have identified a varying number of such sub-groups for a wide range of disorders, including depression (Cuijpers, Van Lier, Van Straten, & Donker, 2005; Gunn et al., 2013; Lutz et al., 2009; Sunderland, Wong, Hilvert-Bruce, & Andrews, 2012), anxiety (Sunderland et al., 2012), panic disorder (Lutz et al., 2014), post-traumatic stress disorder (PTSD) (Elliott, Biddle, Hawthorne, Forbes, & Creamer, 2005; Stein, Dickstein, Schuster, Litz, & Resick, 2012), and first-episode psychosis (Hodgekins et al., 2015).

Previous studies of both therapy outcomes and treatment response trajectories in psychological therapy have some limitations. First, with few exceptions (e.g. Ali et al., 2014; Flückiger, Grosse Holtforth, Znoj, Caspar, & Wampold, 2013; Green, Barkham, Kellett, & Saxon, 2014; Lutz, Martinovich, Lyons, Leon, & Stiles, 2007; Saxon et al., 2017), they have not accounted for the structure of longitudinal data in which patients are nested under individual therapists. Failure to take into account this hierarchical data structure can result in biased statistical inferences (Stochl et al., 2014). Second, many of the previous studies have relied upon specialised, non-routine variables (e.g. measures of therapeutic alliance). Although these provide valuable insights, there is a practical need for easily identifiable, routinely-collected variables, including patients' sociodemographic characteristics and clinical features (Van et al., 2008a).

These limitations highlight a need for robust studies that use appropriately complex multilevel models to cope with hierarchical and longitudinal dependencies, as well as convenient [i.e. readily available in Improving Access to Psychological Therapies' (IAPT) routinely-collected variables] and practical (i.e. relevant to outcomes of interest) variables to explain heterogeneity in treatment response trajectories and symptomatic recovery. In this exploratory analysis, we aimed to identify variables that are associated with (1) baseline symptom severity, (2) rate of symptomatic change, and (3) symptomatic recovery of patients receiving psychological therapy in England's IAPT primary mental health care setting.

## Methods

### Setting

The IAPT programme in England began in 2008 with a direct objective to increase public access to National Institute for Health and Care Excellence (NICE)-approved psychological therapies for depression and anxiety. IAPT currently assesses over 1 300 000 people annually and delivers therapy to approximately 550 000. The programme offers low- (step 2) and high-intensity (step 3) treatments. Low-intensity approaches include guided self-help, psychoeducation, computerised cognitive–behavioural therapy (CBT), behavioural activation, and structured group activity programmes (Clark, 2018). In high-intensity services, face-to-face CBT is the predominant approach, although there is a wider range of recommended treatments (e.g. eye movement desensitisation and reprocessing, interpersonal psychotherapy, counselling for depression, compassion-focused therapy, and integrative counselling). On average, patients receive seven sessions over a period of 3–4 months.

At each session, therapists assess depression and anxiety symptoms using the 9-item Patient Health Questionnaire (PHQ-9) (Kroenke, Spitzer, & Williams, 2001) and the 7-item Generalised Anxiety Disorder assessment (GAD-7) (Spitzer, Kroenke, Williams, & Lowe, 2006), respectively. IAPT services adopted these scales nationally because of their good psychometric properties (Cameron, Crawford, Lawton, & Reid, 2008; Titov et al., 2011) and brevity, and they use them to monitor improvement and recovery rates. Total scores are computed as sum scores of items (response categories: 0 = Not at all; 1 = Several days, 2 = More than half the days; 3 = Nearly every day). PHQ-9 scores range from 0 (no depression) to 27 (severe depression), while GAD-7 scores range between 0 (no anxiety) and 21 (severe anxiety). In IAPT, individuals are described at ‘caseness’, if they score above the clinical cut-off for depression (PHQ-9 ⩾ 10) (Manea, Gilbody, & McMillan, 2012) and/or anxiety (GAD-7 ⩾ 8).

### Participants

The primary sample consisted of 35 527 individuals across Cambridgeshire and Peterborough NHS Foundation Trust and Sussex Partnership NHS Foundation Trust who accessed IAPT services between February 2018 and December 2018. We excluded 6717 individuals deemed not suitable for the service after initial assessment and those with no longitudinal data (i.e. who only attended one appointment). The sample analysed for determinants of baseline symptom severity and rate of symptomatic change consisted of 28 810 individuals (Table 1).

**Table 1.** Sample characteristics and descriptive statistics for variables hypothesised to be associated with outcomes

	Full sample (*n* = 28 810)	Recovery subsample (*n* = 8114)
Gender: count (%)	Females = 19 191 (66.6)	Females = 5378 (66.3)
	Males = 9462 (32.8)	Males = 2736 (33.7)
	Missing = 157 (0.6)	Missing = 0 (0)
Age: mean (s.d.)	41.3 (15.3)	39.4 (13.3)
Functioning (WSAS): mean (s.d.)	19.2 (9.1)	20.0 (8.6)
Socioeconomic status (median IMD): mean (s.d.)	6.3 (2.0)	6.4 (1.9)
Therapy intensity: count (%)	Low = 17 189 (59.7)^a^	Low = 5037 (62.1)
	High = 10 884 (37.8)^a^	High = 3077 (37.9)
	Missing = 737 (2.5)	Missing = 0 (0)
Therapy frequency (gap between therapies in days): median (IQR)	14.0 (12.5)	14.0 (10)
Baseline symptom severity PHQ-9: mean (s.d.)	13.6 (6.3)	14.7 (5.3)
Baseline symptom severity GAD-7: mean (s.d.)	12.6 (5.3)	14.0 (4.1)
Number of patients per therapist: median (IQR)	29 (80.8)	52 (84)
Ethnicity: count (%)	White = 25 254 (87.6)	White = 6538 (80.6)
	Indian = 334 (1.2)	Indian = 131 (1.6)
	Asian = 238 (0.8)	Asian = 86 (1.1)
	Black = 206 (0.7)	Black = 63 (0.8)
	Mixed/other = 717 (2.5)	Mixed/other = 235 (2.9)
	Missing = 2061 (7.2)	Missing = 1061 (13.0)
Diagnosis: count (%)	Depression = 11 194 (38.9)	Depression = 3096 (38.2)
	Anxiety = 9219 (32.0)	Anxiety = 2562 (31.6)
	OCD = 839 (2.9)	OCD = 270 (3.3)
	PTSD = 1166 (4.0)	PTSD = 304 (3.8)
	Panic disorder = 791 (2.7)	Panic disorder = 252 (3.1)
	Phobia = 1084 (3.8)	Phobia = 294 (3.6)
	Other/unspecified = 636 (2.2)	Other/unspecified = 239 (2.9)
	Missing = 3881 (13.5)	Missing = 1097 (13.5)

OCD, obsessive-compulsive disorder; PTSD, post-traumatic stress disorder.

a Of those initially assigned to low or high intensity therapy 9544 individuals (33.1%) were stepped up or down in intensity during the therapy course.

To assess which variables were associated with symptomatic recovery, we analysed therapy outcomes for a subsample of 8114 individuals comprising those patients who (a) had non-missing values for both PHQ-9 and GAD-7 scores at first and last assessment (*n* = 27 835); (b) were considered at ‘caseness’ at initial assessment (i.e. PHQ-9 and/or GAD-7 score was above clinical cut-off, *n* = 20 959); (c) had completed treatment (in any number of sessions, *n* = 10 308^1^†); and (d) had non-missing values on all variables hypothesised to be associated with outcomes (*n* = 8114). Over 67% (5438) achieved symptomatic recovery.

### Outcomes

Our outcomes of interest were *baseline symptom severity*, *rate of symptomatic change*, and *symptomatic recovery*. We inferred each patient's baseline symptom severity and rate of symptomatic change from their growth curve (hereafter denoted as *treatment response trajectory*), which is estimated using total scores on the corresponding scale across therapy appointments. *Symptomatic recovery* in IAPT is achieved when a patient who is at ‘caseness’ at pre-treatment has dropped below the clinical cut-off for *both* depression *and* anxiety post-treatment (i.e. PHQ-9 < 10 *and* GAD-7 < 8) (National Collaborating Centre for Mental Health, 2018).

### Variables tested for association with outcomes

The set of variables tested for associations with our outcomes of interest included three sociodemographic variables (*gender*, *age*, and *socioeconomic status*), two clinical variables (*baseline symptom severity* and *baseline level of functioning*), and three therapeutic variables [*therapy intensity* (low- (step 2) or high-intensity (step 3)), *therapy frequency* (median number of days between sessions), and the number of patients treated by an individual therapist (*caseload*)]. All of these variables were directly obtained or derived from data routinely collected in IAPT services (see online Supplementary Table S1 for a detailed variable list).

*Baseline level of functioning* was measured using the 5-item self-report Work and Social Adjustment Scale (WSAS) (Mundt, Marks, Shear, & Greist, 2002), which measures personal, occupational, and social functional impairment. Each item on the WSAS is scored from 0 (no impairment) to 8 (severe impairment), hence total scores range from 0 to 40, with higher total scores indicating more severe impairment.

*Socioeconomic status* was estimated using the index of multiple deprivation (IMD). IMD deciles are publicly available data for postcodes across the UK. Anonymised data provided by IAPT, however, included only the outward area postcode. The IMD for each individual was therefore estimated as a median IMD decile for the corresponding outward area, with lower values representing higher deprivation.

### Statistical analysis

We used growth models to estimate patients' treatment response trajectories of depression and anxiety symptoms. This modelling approach fits, for each patient, a non-linear trend for the total scores of PHQ-9 or GAD-7 over the course of therapy. We inferred values for baseline symptom severity and rate of symptomatic change from the intercept and slope of patients' treatment response trajectories, respectively. In all analyses we used full information maximum likelihood estimator to account for data missingness.

The IAPT treatment model is based on 12–20 sessions as set out in the NICE guidelines. Very few individuals attended more than 20 appointments and thus we only used the first 20 appointments for each patient. We first estimated the basic nonlinear growth model (with intercept, slope, and quadratic term as latent variables) anchored at every attended appointment (see online Supplementary Fig. S1).

At this stage, we investigated whether the individual treatment trajectories clustered into homogeneous classes using a growth mixture model. If few interpretable classes were found, then the variables associated with baseline symptom severity and rate of symptomatic change could be considered for these classes instead of individual trajectories. However, we did not find such classes (see online Supplementary Appendix 1) and thus carried out the analysis at an individual level.

Next, we reparameterised the model so that the slope represented change over the first seven appointments rather than between the first and the second appointments as in the basic model (note that all 20 appointments were still used to estimate this model). We chose to report the symptomatic change in the first seven appointments because it is the average number of appointments nationally and thus represents a typical length of therapeutic intervention within IAPT (Public Health England, 2019). We detail the model in online Supplementary Appendix 2.

Additional analytical complexity stemmed from the multilevel structure of the data (multiple patients received therapy from the same therapist). Accounting for this analytically provides unbiased treatment response trajectories. In addition, it allowed us to assess variables associated with the average rate of symptomatic change for each therapist's patients. Additional details are provided in online Supplementary Appendix 3.

We then added variables considered to be related to the intercepts (baseline symptom severity) and slopes (rate of symptomatic change) of treatment response trajectories to the reparameterised model. At the patient level (the within-level part of the model), we included gender, age, socioeconomic status, and baseline level of functioning (WSAS). We included therapy intensity (low *v.* high) as an important covariate accounting for different therapeutic approaches applied for low- and high-intensity IAPT patients. Therapy frequency was considered only in the context of the slope as its association with the intercept would be conceptually non-sensical. We also explored the association between baseline symptom severity and rate of symptomatic change. At the therapist level (the between-level part of model), we examined whether caseload was associated with (a) the average rate of symptomatic change and (b) recovery for each therapist's patients.

We used a multilevel logit model for variables associated with the binary clinical endpoint of symptomatic recovery. The set of variables examined in relation to symptomatic recovery was identical to that of rate of symptomatic change except that (a) we included baseline symptom severity of both depression and anxiety (as recovery in IAPT requires having scores below corresponding threshold on both measures) and (b) these baseline symptom severities were operationalised as total scores of PHQ-9 and GAD-7 at initial assessment (i.e. not as intercepts of treatment response trajectories). Total PHQ-9 and GAD-7 scores at baseline were moderately correlated (*r* = 0.46), allowing inclusion of both variables in the same model. We bootstrapped the model (1000 iterations) to obtain bootstrapped confidence intervals (CIs) for odds ratios. We conducted all analyses in MPlus 8.4 (Muthén & Muthén, 1998-2019) and R 3.6.3 (R Core Team, 2019).

The script for our analyses and synthetic data are available at https://osf.io/48eur/.

## Results

### Sample and variable descriptives

Table 1 provides the sociodemographic characteristics and basic descriptive statistics of variables hypothesised to be associated with outcomes.

### Growth models

**Fig. 1.**
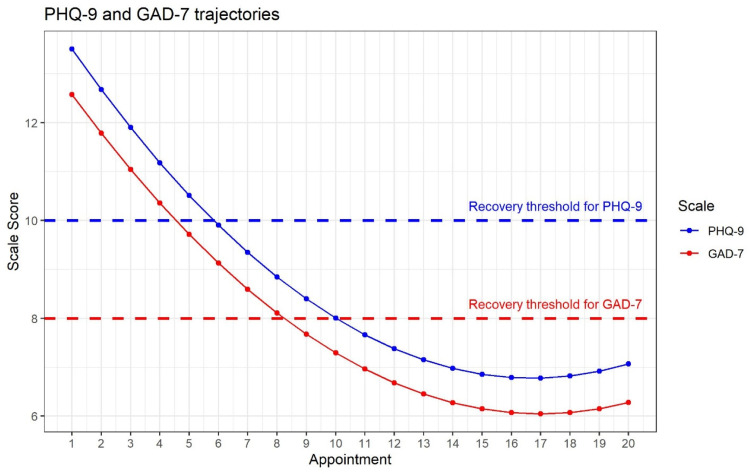
Estimated average growth model trajectories for PHQ-9 (blue) and GAD-7 (red). Scale score is computed as sum score of all items.

The basic nonlinear growth models fit the data well (PHQ-9: RMSEA = 0.034, CFI = 0.952, TLI = 0.955, SRMR = 0.082; GAD-7: RMSEA = 0.032, CFI = 0.951, TLI = 0.954, SRMR = 0.080). Figure 1 depicts the estimated mean trajectories for the two scales. The modelled mean baseline symptom severity (intercept) had a value of 13.5 for the PHQ-9 and 12.6 for the GAD-7. The mean slopes (−0.9 for the PHQ-9 and −0.8 for the GAD-7) reflect the average change in scores between the first and second therapy sessions. Such interpretation is not very informative with regards to understanding improvement over the course of the therapy. In the reparameterised growth model, the slopes represent change over the first seven sessions. Results suggest that the average improvement across seven therapy sessions is 4.2 points on the PHQ-9 and 4.0 points on the GAD-7 (see online Supplementary Appendix 2 for details).

### Variables associated with baseline symptom severity and rate of symptomatic change

Tables 2 and 3 include estimated regression coefficients (including standardised estimates) for depression and anxiety symptoms, respectively.

**Table 2.** Regression coefficients for conditional multilevel growth model of depressive symptoms

Level	Outcome	Independent variable^a^	Estimate (standard error)	Standardised estimate (standard error)	*p* value
Individual level (within)	BSS	Gender^b^	−0.401 (0.081)	−0.034 (0.007)	<0.001
	BSS	Age	−0.021 (0.003)	−0.051 (0.007)	<0.001
	BSS	Functioning (WSAS)	0.337 (0.005)	0.547 (0.007)	<0.001
	BSS	Socioeconomic status (median IMD)	−0.177 (0.026)	−0.061 (0.009)	<0.001
	BSS	Therapy intensity	0.374 (0.103)	0.032 (0.009)	<0.001
	RoSCh	Gender^b^	−0.190 (0.073)	−0.025 (0.010)	0.009
	RoSCh	Age	−0.017 (0.003)	−0.063 (0.011)	<0.001
	RoSCh	Functioning (WSAS)	0.022 (0.005)	0.055 (0.013)	<0.001
	RoSCh	Socioeconomic status (median IMD)	0.068 (0.032)	0.037 (0.017)	0.037
	RoSCh	Therapy intensity	−0.005 (0.090)	−0.001 (0.012)	0.960
	RoSCh	Therapy frequency	0.002 (0.006)	0.010 (0.026)	0.681
	RoSCh	Baseline symptom severity (Intercepts within)	−0.252 (0.013)	−0.393 (0.014)	<0.001
Therapist level (between)	RoSCh	Number of patients	−0.005 (0.003)	−0.319 (0.153)	0.037

RoSCh, rate of symptomatic change (where more negative slopes indicate more rapid improvement); BSS, baseline symptom severity.

*R*^2^: RoSCh = 0.138, BSS = 0.321.

a All independent variables are cross-adjusted.

b Reference group = females.

**Table 3.** Regression coefficients for conditional multilevel growth model of anxiety symptoms

Level	Outcome	Independent variable^a^	Estimate (standard error)	Standardised estimate (standard error)	*p* value
Individual level (within)	BSS	Gender^b^	−0.937 (0.072)	−0.095 (0.007)	<0.001
	BSS	Age	−0.036 (0.003)	−0.102 (0.008)	<0.001
	BSS	Functioning (WSAS)	0.207 (0.004)	0.403 (0.008)	<0.001
	BSS	Socioeconomic status (median IMD)	−0.115 (0.024)	−0.048 (0.010)	<0.001
	BSS	Therapy intensity	−0.193 (0.097)	−0.020 (0.010)	0.048
	RoSCh	Gender^b^	−0.092 (0.063)	−0.014 (0.010)	0.145
	RoSCh	Age	−0.014 (0.003)	−0.058 (0.010)	<0.001
	RoSCh	Functioning (WSAS)	0.044 (0.004)	0.126 (0.012)	<0.001
	RoSCh	Socioeconomic status (median IMD)	0.013 (0.024)	0.008 (0.015)	0.577
	RoSCh	Therapy intensity	0.005 (0.088)	0.001 (0.013)	0.955
	RoSCh	Therapy frequency	0.008 (0.005)	0.042 (0.025)	0.091
	RoSCh	Baseline symptom severity (Intercepts within)	−0.262 (0.012)	−0.388 (0.012)	<0.001
Therapist level (between)	RoSCh	Number of patients	−0.006 (0.002)	−0.322 (0.092)	0.001

RoSCh, rate of symptomatic change (where more negative slopes indicate more rapid improvement); BSS, baseline symptom severity.

*R*^2^: RoSCh = 0.127, BSS = 0.188.

a All independent variables are cross-adjusted.

b Reference group = females.

#### Variables associated with baseline symptom severity and rate of symptomatic change for depression (PHQ-9)

Patients' modelled baseline symptom severity (the within-level intercept of their treatment response trajectory) was significantly related to gender (females have greater baseline symptom severity), age (younger patients have greater baseline symptom severity), baseline functioning (patients with more functional impairment have greater baseline symptom severity), socioeconomic status (patients living in areas of higher deprivation have greater baseline symptom severity), and therapy intensity. The significant positive relationship between baseline symptom severity and therapy intensity confirms that patients with more severe depression symptoms tend to be assigned to high-intensity therapy. In terms of standardised coefficients, baseline symptom severity had the strongest relationship with baseline functioning scores.

Patients' rate of symptomatic change for depression (the within-level slope of patients' treatment response trajectories) was most strongly related (in terms of magnitude of impact) to their baseline depression severity. More specifically, the higher the baseline symptom severity, the faster the improvement. Additionally, the rate of symptomatic change was related to gender (males improve more rapidly), age (older patients improve more rapidly), baseline level of functioning (patients with less functional impairment improve more rapidly), and socioeconomic status (patients living in areas of higher deprivation improve more rapidly). The improvement rate was *not* significantly related to therapy frequency or intensity.

The average improvement of a particular therapist's patients (the between-level part of the model), was significantly related to that therapist's caseload, however, in an unexpected direction – a larger caseload was related to more rapid improvement.

#### Variables associated with baseline symptom severity and rate of symptomatic change for anxiety (GAD-7)

Results for the GAD-7 treatment response trajectories were similar to those of the PHQ-9. However, contrary to the PHQ-9 results, the relationship between baseline symptom severity and therapy intensity was at the borderline of statistical significance and was in the opposite direction than for depression. Furthermore, gender and socioeconomic status were not related to the rate of improvement for GAD-7 scores. Finally, considering the standardised coefficients, functioning had a larger effect size for anxiety than for depression (with lower impairment related to more rapid improvement), when controlling for all other variables.

### Variables associated with symptomatic recovery

Table 4 shows results of a multilevel logit model with symptomatic recovery as the outcome. Baseline symptom severity, age, and baseline level of functioning were significantly related to symptomatic recovery, even when bootstrap was applied. Specifically, with each additional point on the PHQ-9 or GAD-7 at the beginning of therapy, the chances of symptomatic recovery decrease by approximately 5.8% and 6.3%, respectively. Similarly, an increase of one point on the WSAS is associated with an approximate 2.4% reduction in chances of recovery. Each additional year of age increases odds of recovery by approximately 0.8%.

**Table 4.** Regression coefficients, odds ratios and bootstrapped odds ratios for variables hypothesised to be associated with symptomatic recovery

Level	Independent variable^a^	Estimate (standard error)	Odds ratio	*p* value	Bootstrapped odds ratios mean (95% CI)
Individual level (within)	Baseline symptom severity PHQ-9	−0.060 (0.007)	0.942	<0.001	0.940 (0.922–0.958)
	Baseline symptom severity GAD-7	−0.065 (0.007)	0.937	<0.001	0.935 (0.916–0.953)
	Gender	0.076 (0.049)	1.079	0.138	1.090 (0.947–1.248)
	Age	0.008 (0.002)	1.008	<0.001	1.008 (1.003–1.014)
	Functioning (WSAS)	−0.024 (0.003)	0.976	<0.001	0.975 (0.966–0.985)
	Therapy frequency	−0.008 (0.003)	0.992	0.015	0.994 (0.985–1.003)
	Therapy intensity	−0.119 (0.060)	0.888	0.034	0.886 (0.748–1.048)
	Socioeconomic status (median IMD)	−0.021 (0.016)	0.979	0.180	0.985 (0.941–1.033)
Therapist level (between)	Number of patients	0.000 (0.001)	1.000	0.446	1.001 (0.999–1.002)

a All independent variables were cross-adjusted.

Therapy intensity and frequency were significantly related to symptomatic recovery; however, bootstrapped odds ratios were not and thus the results should be interpreted with caution. In addition, both variables have a relatively small effect on recovery. For example, an increase of a day in the median number of days between sessions reduced the probability of recovery by 0.7% and being assigned to high-intensity therapy lowered chances of recovery by approximately 11.2% when adjusting for all other variables (it is important to note, however, that high-intensity therapists generally see more complex and severe patients).

Socioeconomic status, gender, and therapist caseload were not significantly associated with symptomatic recovery.

The results for the sample including those individuals who had not yet completed therapy or who had dropped out (whose symptomatic recovery was derived from the last recorded session) are presented in online Supplementary Appendix 4. They were very similar to those presented in Table 4.

## Discussion

In this study, we explored which sociodemographic, clinical, and therapeutic variables may be related to (1) baseline symptom severity, (2) rate of symptomatic change, and (3) symptomatic recovery (as defined by IAPT criteria) for patients with depression and/or anxiety engaging in psychological therapy in IAPT services. Importantly, our predictors were all variables that are routinely collected in IAPT services, including individual variables (gender, age, andsocioeconomic status), clinical variables (baseline depression and anxiety scores, and baseline level of functioning), and therapeutic variables [therapy intensity and median number of days between sessions (i.e. frequency)]. Our multilevel approach also allowed us to test the relationship between therapists' IAPT caseload and the average rate of symptomatic change and recovery of their corresponding patients.

### Treatment response trajectories

Although baseline severity of both depression and anxiety symptoms was significantly related to all included variables (i.e. gender, age, baseline functioning, socioeconomic status, and therapy intensity), baseline functioning had by far the largest effect sizes (whereby greater functional impairment was associated with more severe symptomatology). Age, baseline functioning, baseline symptom severity, and therapist caseload were significantly related to the rate of change for both depression and anxiety symptoms, and gender and socioeconomic status were additionally related to rate of change for depression (but not anxiety) symptoms. Therapy characteristics such as intensity and frequency seemed to have no relation to the rate of symptomatic change. Baseline symptom severity was the most important variable associated with rate of change for both depression and anxiety symptoms, whereby patients with more severe symptomatology at the start of therapy improved more rapidly. On average, a 1-point difference in total score on the corresponding scale between two patients at baseline results in an expected difference of 0.748 (i.e. 1 − 0.252) points on the PHQ-9 and 0.738 (i.e. 1 − 0.262) points on the GAD-7 between them at seventh session. Baseline functioning was the only other variable to show a clinically-relevant effect size at the individual level (whereby greater functional impairment at the start of therapy was related to slower improvement), but only for anxiety symptoms.

Our finding that greater baseline severity of depression and anxiety symptoms was associated with more rapid symptom improvement is unusual. In general, others have found either that baseline severity negatively impacts the rate of symptomatic improvement [e.g. Sunderland et al.'s (2012) study of online CBT for depression and anxiety disorders] or has no relationship [e.g. Comninos & Grenyer's (2007) study of early rapid response in supportive-expressive dynamic psychotherapy for major depression]. The importance of baseline symptom severity extends beyond symptomatic change, as Hodgekins et al. (2015) demonstrated in their finding that baseline severity of psychotic symptoms serves as a predictor of belonging to a ‘poorer’ trajectory of social recovery for patients with first episode psychosis.

We located only one study that reflected our finding that baseline severity was positively related to the rate of symptomatic improvement [Elliott et al.'s (2005) study of veterans receiving treatment for PTSD]. It is conceivable that help-seeking individuals with higher baseline symptom severity may be more motivated to engage with therapy in an effort to overcome more severe symptoms ('the gift of desperation’) and that increased engagement positively impacts treatment outcomes (Dixon et al., 2016). However, this explanation is perhaps more reasonable for anxiety symptoms than depression symptoms, wherein greater severity may instead be a barrier to engagement. Furthermore, it is important to consider the potential for statistical artefacts when interpreting these effect sizes, as patients starting with more severe symptoms have greater scope for improvement.

Our finding about the negative impact of functional impairment on rate of symptomatic improvement is more consistent with the literature. For example, Lutz et al.'s (2014) study of CBT for patients with panic disorder highlighted the importance of social functioning in predicting class membership (as characterised in part by rate of symptomatic change). Poor functioning is a well-documented barrier to symptomatic improvement. Many people with greater functional impairment are unemployed and/or have fewer social contacts, and thus are missing two key protective factors associated with positive mental health. Poor functioning is further related to barriers in engaging with therapy. For example, poorer functioning may equate to fewer resources for use in therapy or poor attendance (due to various reasons including financial or social difficulties and anxiety). Thus, interventions for those with poorer social functioning may require additional considerations, such as strategies for returning to work or building up social networks and overcoming barriers related to each of these goals (Knight et al., 2020).

At the therapist level, our finding that a larger therapist caseload was associated with more rapid improvement could indicate that more frequent application of IAPT techniques facilitates greater therapist competency. Alternatively, this could simply indicate that more competent therapists are assigned more patients. In either case, this result should not be interpreted causally. First, the caseload variable represents only the number of *IAPT* patients seen by each therapist and is not weighted for the number of days worked in IAPT. It is possible that each therapist sees additional patients outside of IAPT, in which case the effect of *total* caseload would be unmeasured in our analyses. Second, some IAPT therapists focus on specific groups of patients (e.g. patients with long-term physical conditions), which could affect their caseload and potentially bias results; however, this specialisation applies to a relatively small group of therapists.

### Symptomatic recovery

Higher chance of symptomatic recovery, as defined by IAPT criteria, was associated with lower baseline severity of depression and anxiety symptoms, lower functional impairment, and increased age, with baseline symptom severity having the greatest effect. These findings are not particularly surprising: while starting therapy with a higher score on the PHQ-9/GAD-7 enables more scope for improvement (hence the sensibility of its association with faster symptomatic improvement), it also implies a further distance to IAPT's recovery threshold. Several other studies have found similar results in terms of baseline symptom severity (Amati et al., 2018; Marttunen et al., 2008), including two within the IAPT setting (Green et al., 2015; Gyani et al., 2013a, 2013b). These patients may be at increased risk for additional psychiatric comorbidities, including psychotic experiences (Stochl et al., 2015), which could further contribute to the reduced chance of recovery (Knight et al., 2020).

In interpreting our results, it must be acknowledged that people with ‘less favourable’ characteristics (e.g. those with higher baseline symptom severity/functional impairment) do not necessarily benefit *less* from therapy. It is important to remember that the definition of symptomatic recovery (as routinely used in IAPT for performance monitoring purposes) is centred around *absolute improvement* (i.e. whether their symptoms were reduced beyond the recovery ‘threshold’) rather than *relative improvement* (i.e. the difference between baseline and final symptom severity). Hence, in order to be most informative, results about symptomatic recovery should be contextualised within our discussion of treatment response trajectory.

### Strengths and limitations

The main strength of this study is the large clinical sample. Furthermore, although this is not the first study that has explored variables related to symptomatic improvement and recovery in the context of psychological therapy, we used an analytical approach that is more appropriate for the complex data structure of longitudinal outcomes nested under therapists. A further strength is the applicability to clinical settings in general, and to the IAPT setting in particular, as we used routinely-collected, easily-obtained measures in our analyses. However, this may also be considered as a weakness, as many potentially relevant variables were not available for inclusion in our analyses, including medication use, treatment history, therapist competence, and therapeutic alliance, as well as other key risk and protective factors. The absence of such prognostic variables can be seen in the relatively low *R*^2^ values for our models.

We acknowledge additional limitations in terms of sample selection and available variables. The selection of referrals meeting IAPT service criteria for treatment may have introduced Berkson's bias into our analyses, particularly in those regarding recovery. We were unable to quantify this bias because we have no recovery data for referrals not admitted to IAPT. Furthermore, the subsample of 8114 with the requisite data for evaluation of predictors of recovery may not be representative of the full sample as patients drop out for non-random reasons; again, this is inherent in many clinical samples where drop-outs may be due to recovery or worsening of symptoms. Therefore, inferences from these findings need to be made with caution. Finally, although our sample size was large, it represented only two mental health trusts, which may limit the generalisability of our results.

In terms of limitations relating to individual variables, we could not investigate relationship between ethnicity and treatment response trajectories as we had very few Black, Asian, and Minority Ethnic (BAME) individuals in our sample (although this broadly reflects the proportion of BAME patients accessing IAPT nationally). Although beyond the scope of this paper, the low proportion of BAME individuals accessing IAPT services merits careful consideration. Low participation may be due to a number of causes, including individual factors (e.g. personal attitudes towards services), service-level factors (e.g. inaccessibility or unacceptability), and wider cultural issues (e.g. discrimination and stigma). Furthermore, our calculation of socioeconomic status has limitations; as IAPT does not collect this information on an individual level, we derived this variable by using the outward code of each individual's postcode to calculate the median IMD decile for the area, which may not be representative of an individual's experience. Finally, in terms of caseload, we were only able to identify the number of patients the therapist sees *within the IAPT setting*. Yet, it is not uncommon for IAPT therapists to see additional patients outside of IAPT.

## Conclusions

Therapist confidence and self-efficacy are important factors for determining therapy effectiveness (Green et al., 2014; Heinonen, Lindfors, Laaksonen, & Knekt, 2012). Equally as important, patients' positive *expectations* of therapy outcome have been consistently linked to better *actual* outcomes (Mondloch, Cole, & Frank, 2001). Although therapists and patients may worry about progress and outcomes in the context of more severe baseline symptomatology, our findings suggest that they can take courage in the knowledge that more ‘unwell’ patients actually have the potential to improve more rapidly. Moreover, this finding demonstrates the gains possible for patients with more severe depression and anxiety in services offering short-term psychological therapies.

Furthermore, our results regarding variables associated with symptomatic recovery are useful for highlighting groups of patients that may benefit from additional or more intensive intervention. One such group consists of patients who begin therapy with more severe depression and/or anxiety symptoms and poorer functioning, as these two characteristics have a significant negative impact on symptomatic recovery. In order to ensure that everyone has the potential to reach the IAPT symptomatic recovery threshold, we must continue to investigate how to best tailor interventions to fit individual patients' needs.

## Footnotes

† The notes appear after the main text.

## Note

1 In response to a suggestion following peer review, we have also analysed the sample that included individuals who had not completed treatment and whose symptomatic recovery was derived from the last recorded session. The results are presented in Appendix 4.

## Supplementary material

For supplementary material accompanying this paper visit https://doi.org/10.1017/S0033291720005395. click here to view supplementary material
