# Supplementary material for: Determinants of patient-reported outcome trajectories and symptomatic recovery in Improving Access to Psychological Therapies (IAPT) services
Source: Psychol Med. 2021 Mar 8;52(14):3231–40. doi: 10.1017/S0033291720005395 (PMC9693716; doi:10.1017/S0033291720005395)
Supplement: Supplementary file 1 [file S0033291720005395sup001.zip › S0033291720005395sup001.docx]

***Appendix 1: Classes of treatment response trajectories.***

A growth mixture model with 1-9 classes was fitted to the data to establish if treatment response trajectories cluster into interpretable homogeneous classes. The fit of the estimated models is presented in Supplementary Table 1. Dropping indices such as Bayesian Information Criterion (BIC), Akaike Information Criterion (AIC) in combination with entropy values do not support few homogeneous, yet distinct classes.

**Supplementary Table 1: Fit of growth mixture model with 1-9 classes.**

|  | **Title** | **Observations** | **Number of parameters** | **Log-Likelihood** | **AIC** | **BIC** | **Entropy** |
| --- | --- | --- | --- | --- | --- | --- | --- |
| PHQ-9 | 1-class | 27835 | 23 | -555535 | 1111117 | 1111306 | - |
|  | 2-classes | 27835 | 27 | -514358 | 1028771 | 1028993 | 0.828 |
|  | 3-classes | 27835 | 31 | -498217 | 996497 | 996752 | 0.801 |
|  | 4-classes | 27835 | 35 | -491410 | 982890 | 983178 | 0.773 |
|  | 5-classes | 27835 | 39 | -488228 | 976534 | 976855 | 0.743 |
|  | 6-classes | 27835 | 43 | -485876 | 971839 | 972193 | 0.723 |
|  | 7-classes | 27835 | 47 | -484107 | 968307 | 968694 | 0.712 |
|  | 8-classes | 27835 | 51 | -482741 | 965585 | 966005 | 0.688 |
|  | 9-classes | 27835 | 55 | -481639 | 963387 | 963840 | 0.681 |
| GAD-7 | 1-class | 27832 | 23 | -531038 | 1062122 | 1062311 | - |
|  | 2-classes | 27832 | 27 | -493235 | 986523 | 986745 | 0.816 |
|  | 3-classes | 27832 | 31 | -479348 | 958757 | 959012 | 0.78 |
|  | 4-classes | 27832 | 35 | -473553 | 947177 | 947465 | 0.746 |
|  | 5-classes | 27832 | 39 | -471127 | 942332 | 942653 | 0.719 |
|  | 6-classes | 27832 | 43 | -468636 | 937358 | 937713 | 0.699 |
|  | 7-classes | 27832 | 47 | -467019 | 934131 | 934518 | 0.676 |
|  | 8-classes | 27832 | 51 | -465694 | 931491 | 931911 | 0.669 |
|  | 9-classes | 27832 | 55 | -464716 | 929543 | 929995 | 0.660 |
