# Supplementary material for: Determinants of patient-reported outcome trajectories and symptomatic recovery in Improving Access to Psychological Therapies (IAPT) services
Source: Psychol Med. 2021 Mar 8;52(14):3231–40. doi: 10.1017/S0033291720005395 (PMC9693716; doi:10.1017/S0033291720005395)
Supplement: Supplementary file 1 [file S0033291720005395sup001.zip › S0033291720005395sup002.docx]

***Appendix 2: Reparameterised model***

The traditional parameterisation of growth models is useful for estimating the shape of treatment response trajectories but limits the interpretation of the slope as the average improvement in scores between the first and second therapy session when the trajectory shape is, as in our case, nonlinear. Fortunately, the model can be reparameterised such that the slope can be interpreted as growth between two arbitrarily-chosen appointments. Times for the appointments are estimated for all except for the anchoring appointments (i.e. the anchor appointments are fixed), so that the. Non-linearity of recovery trajectories is still taken into account. In our case, we have chosen the 1^st^ and the 7^th^ appointments as our anchoring appointments. Therefore, the slope can be interpreted as the *difference in modelled scores between these two appointments*. The result is a linear model, although the non-linearity resulting from differential changes in symptoms between appointments is still taken into account. The fit of this model is slightly worse compared to non-reparameterised model, yet still acceptable for both the PHQ-9 (RMSEA=0.042, RMSEA 90% CI=(0.042-0.043), CFI=0.923, TLI=0.922, SRMR=0.145) and the GAD-7 (RMSEA=0.042, RMSEA 90% CI=(0.041-0.043), CFI=0.929, TLI=0.922, SRMR=0.183). The treatment response trajectories of the reparameterised model are depicted in Supplementary Figure 2). The slope of the reparameterised growth model has a value of -4.208 for the PHQ-9 and of -4.005 for the GAD-7. This suggests that, on average, patients improve by about 4.2 points on PHQ-9 score, and by 4 points on GAD-7 score, over the course of 7 appointments. The correlation between slope and the intercept was negative (-0.331 for PHQ-9 and -0.311 for GAD-7), suggesting that individuals who are initially more depressed and anxious tend to improve faster on the corresponding scale.


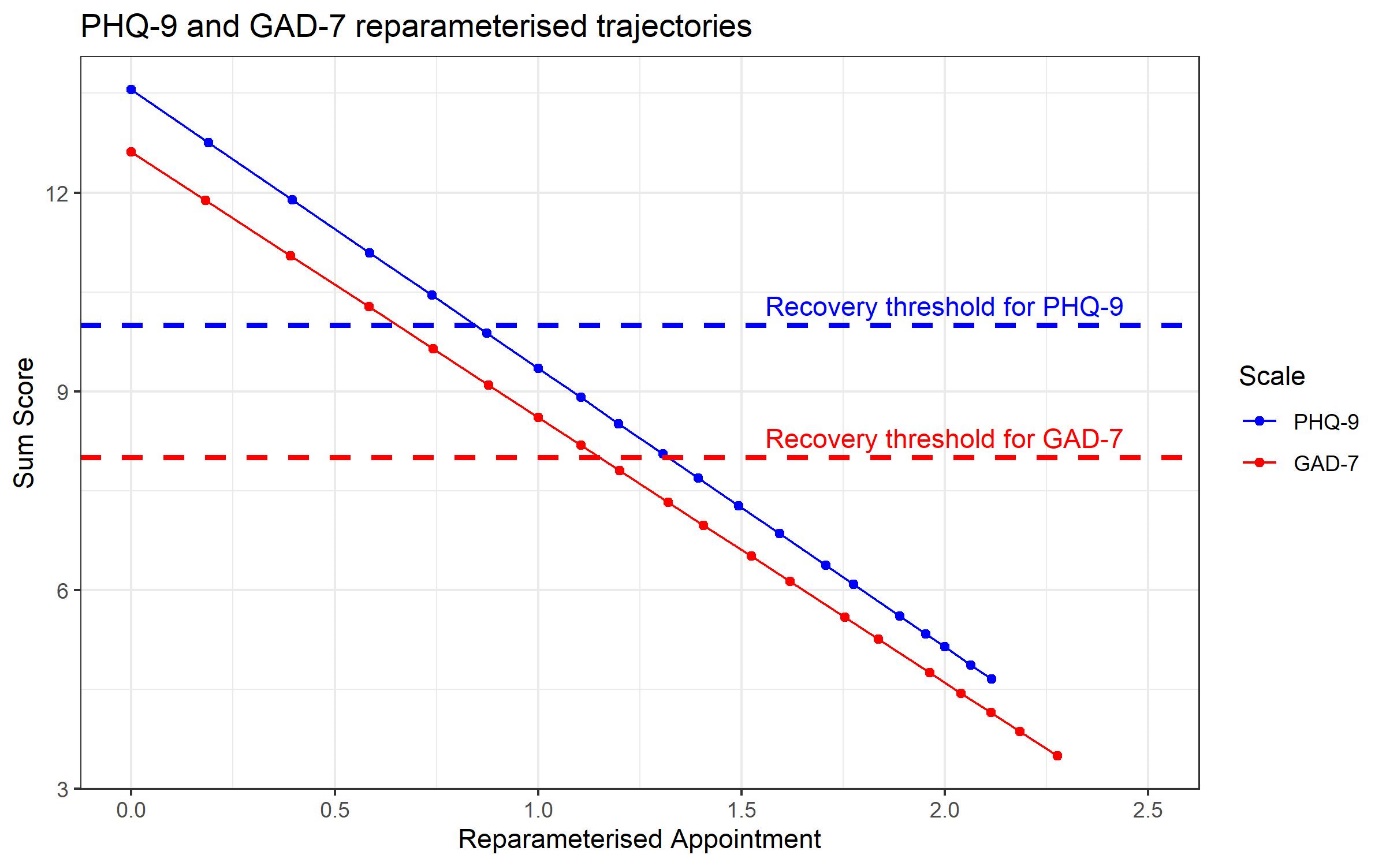


**Supplementary Figure 2:** Estimated growth model trajectories for the reparameterised growth model (PHQ-9 in blue and GAD-7 in red)
