# Supplementary material for: Determinants of patient-reported outcome trajectories and symptomatic recovery in Improving Access to Psychological Therapies (IAPT) services
Source: Psychol Med. 2021 Mar 8;52(14):3231–40. doi: 10.1017/S0033291720005395 (PMC9693716; doi:10.1017/S0033291720005395)
Supplement: Supplementary file 1 [file S0033291720005395sup001.zip › S0033291720005395sup003.docx]

***Appendix 3: Multilevel modelling considerations***

Here we have now investigated the amount of variance accounted for by the therapist and site levels in our outcomes of interest (slopes and intercepts of growth curves and recovery). For this we used the intraclass correlation coefficient (ICC) and design effect (DEFF) coefficient. ICCs > 0.05 (5%) and DEFFs > 2 indicate a need for multilevel modelling at the corresponding level (Lai & Kwok, 2015; Muthen & Satorra, 1995). DEFF is inappropriate for very small or very large cluster sizes (Lai & Kwok, 2015) and thus we do not report it for sites (where the average cluster size is of thousands). Results are presented in the table below and suggest that using a two-level model is appropriate for this dataset.

**Supplementary Table 2: Intraclass correlation coefficients and design effects for therapist and site levels.**

| **Level** | **Outcome** | **Intraclass correlation coefficient**  **(% of total outcome variance)** | **Average cluster size** | **Design effect** |
| --- | --- | --- | --- | --- |
| Therapist | Slopes (rates of symptomatic change) | 0.051 (5.1%) | 53.73 | 3.67 |
|  | Intercepts (Baseline symptom severity) | 0.090 (9.0%) | 53.73 | 5.76 |
|  | Recovery | 0.056 (5.6%) | 19.60 | 2.03 |
| Site | Slopes (rates of symptomatic change) | 0.003 (0.3%)* | 13,916 | - |
|  | Intercepts (Baseline symptom severity) | 0.005 (0.5%)* | 13,916 | - |
|  | Recovery | -** | 4,057 | - |

* only 2 clusters (sites) available. Interpret with caution

** estimation failed – likely as a combination of binary outcome (recovery) and 2 clusters (sites)
