# Supplementary material for: Determinants of patient-reported outcome trajectories and symptomatic recovery in Improving Access to Psychological Therapies (IAPT) services
Source: Psychol Med. 2021 Mar 8;52(14):3231–40. doi: 10.1017/S0033291720005395 (PMC9693716; doi:10.1017/S0033291720005395)
Supplement: Supplementary file 1 [file S0033291720005395sup001.zip › S0033291720005395sup004.docx]

***Appendix 4: Variables associated with symptomatic recovery for the more inclusive sample (n=13,349)***

This additional analysis presents the results of the recalculation of our recovery model for a sample that consists of n=8,114 individuals who completed treatment (as presented in the main paper) as well as an additional n=5,235 individuals who a) had not yet completed treatment or had dropped out, b) had attended at least 3 therapy sessions, and c) had both PHQ-9 and GAD-7 scores available at their last recorded session. Their symptomatic recovery status was derived from their PHQ-9 and GAD-7 scores at the last recorded session such that they had “recovered” if both scores were below IAPT recovery thresholds (PHQ-9 < 10 and GAD-7 < 7). The regression coefficients (and odds ratios) are very similar to those presented in the main text (see Table 4). Standard errors and p-values were generally smaller in this more inclusive sample because of the larger sample size.

**Supplementary Table 3: Regression coefficients, odds ratios and bootstrapped odds ratios for variables hypothesised to be associated with symptomatic recovery for the more inclusive sample (n=13,349)**

| Level | Independent variable* | Estimate (standard error) | Odds ratio | p-value | Bootstrapped odds ratios mean  (95% CI) |
| --- | --- | --- | --- | --- | --- |
| **Individual level (within)** | Baseline symptom severity PHQ-9 | -0.074 (0.005) | 0.929 | <0.001 | 0.939 (0.920-0.958) |
|  | Baseline symptom severity GAD-7 | -0.062 (0.005) | 0.940 | <0.001 | 0.935 (0.914-0.956) |
|  | Gender | 0.128 (0.039) | 1.136 | 0.001 | 1.069 (0.905-1.242) |
|  | Age | 0.014 (0.001) | 1.015 | <0.001 | 1.008 (1.002-1.015) |
|  | Functioning (WSAS) | -0.025 (0.003) | 0.975 | <0.001 | 0.975 (0.965-0.986) |
|  | Therapy frequency | -0.031 (0.002) | 0.969 | <0.001 | 0.992 (0.982-1.001) |
|  | Therapy intensity | -0.110 (0.050) | 0.896 | 0.027 | 0.862 (0.724-1.036) |
|  | Socioeconomic status (median IMD) | -0.015 (0.013) | 0.986 | 0.258 | 0.981 (0.934-1.031) |
| Therapist level (between) | Number of patients | 0.000 (<0.001) | 1.000 | 0.750 | 1.000 (0.999-1.002) |

* All independent variables were cross-adjusted
