# Supplementary material for: Determinants of patient-reported outcome trajectories and symptomatic recovery in Improving Access to Psychological Therapies (IAPT) services
Source: Psychol Med. 2021 Mar 8;52(14):3231–40. doi: 10.1017/S0033291720005395 (PMC9693716; doi:10.1017/S0033291720005395)
Supplement: Supplementary file 1 [file S0033291720005395sup001.zip › S0033291720005395sup005.docx]

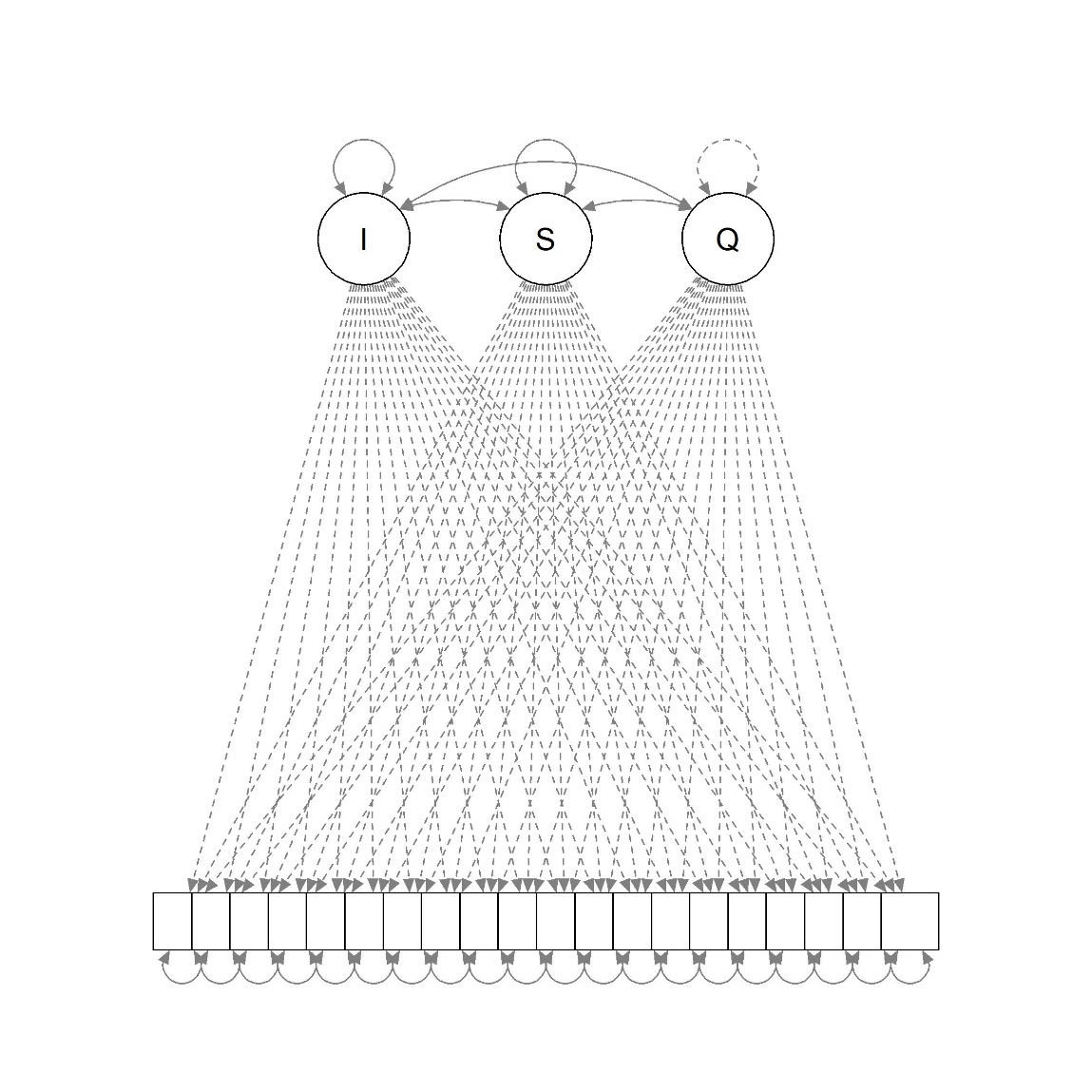


**Supplementary Figure 1:** Conceptual path diagram of unconditional nonlinear growth model. Squares represent observed scores (PHQ-9 or GAD-7) at first 20 appointments, ovals represent latent intercepts (I), slopes (S) and quadratic terms (Q). Dashed arrows show fixed parameters.
