# Supplementary material for: Determinants of patient-reported outcome trajectories and symptomatic recovery in Improving Access to Psychological Therapies (IAPT) services
Source: Psychol Med. 2021 Mar 8;52(14):3231–40. doi: 10.1017/S0033291720005395 (PMC9693716; doi:10.1017/S0033291720005395)
Supplement: Supplementary file 1 [file S0033291720005395sup001.zip › S0033291720005395sup006.docx]

**Supplementary Table 1: List of available variables routinely collected in IAPT and derived variables. Variables in bold were used as predictors, variables in italics were used to derive additional variables.**

| *Available variables routinely collected in IAPT* | *Derived variables* |
| --- | --- |
| **Therapy intensity** | **Age** (derived from Year and Month of birth) |
| ***PHQ-9*** | **IMD** (derived from Postcode) |
| ***GAD-7*** | **Therapy frequency** (derived from Appointment dates) |
| **WSAS (at baseline)** | **Number of patients** (derived from Therapist ID) |
| **Gender** | Recovery (derived from PHQ-9, GAD-7, Appointment number, and End of care reason) |
| *Postcode*  (we had access to outward area portion of postcode) |  |
| *Therapist ID* |  |
| *Appointment Date* |  |
| *Appointment number* |  |
| *Month of birth* |  |
| *Year of birth* |  |
| *End of care reason* |  |
| End of care date |  |
| Ethnicity |  |
| Referral date |  |
| Site |  |
| Service code |  |
| Religion |  |
| Sexuality |  |
| Problem descriptor |  |
| Service research |  |
